# Postsynaptic potentiation of corticospinal projecting neurons in the anterior cingulate cortex after nerve injury

**DOI:** 10.1186/1744-8069-10-33

**Published:** 2014-06-03

**Authors:** Tao Chen, Kohei Koga, Giannina Descalzi, Shuang Qiu, Jian Wang, Le-Shi Zhang, Zhi-Jian Zhang, Xiao-Bin He, Xin Qin, Fu-Qiang Xu, Ji Hu, Feng Wei, Richard L Huganir, Yun-Qing Li, Min Zhuo

**Affiliations:** 1 Center for Neuron and Disease, Frontier Institute of Science and Technology, Xi’an Jiaotong University, Xi'an, China; 2 Department of Anatomy, Histology and Embryology and K.K. Leung Brain Research Center, the Fourth Military Medical University, Xi’an 710032, China; 3 Department of Physiology, Faculty of Medicine, Center for the Study of Pain, University of Toronto, 1 King’s College Circle, Toronto, Ontario M5S 1A8, Canada; 4 Wuhan Institute of Physics and Mathematics, the Chinese Academy of Sciences, Wuhan 430071, China; 5 Wuhan National Laboratory for Optoelectronics, Wuhan 430074, China; 6 College of Life Science and Technology, Huazhong University of Science and Technology, Wuhan 430074, China; 7 University of Chinese Academy of Sciences, Beijing 100049, China; 8 College of Life Science, Wuhan University, Wuhan 430071, China; 9 Department of Biomedical Sciences, University of Maryland Dental School, Baltimore, MD 21201, USA; 10 Department of Neuroscience and Howard Hughes Medical Institute, Johns Hopkins University School of Medicine, Baltimore, MD, USA

## Abstract

Long-term potentiation (LTP) is the key cellular mechanism for physiological learning and pathological chronic pain. In the anterior cingulate cortex (ACC), postsynaptic recruitment or modification of AMPA receptor (AMPAR) GluA1 contribute to the expression of LTP. Here we report that pyramidal cells in the deep layers of the ACC send direct descending projecting terminals to the dorsal horn of the spinal cord (lamina I-III). After peripheral nerve injury, these projection cells are activated, and postsynaptic excitatory responses of these descending projecting neurons were significantly enhanced. Newly recruited AMPARs contribute to the potentiated synaptic transmission of cingulate neurons. PKA-dependent phosphorylation of GluA1 is important, since enhanced synaptic transmission was abolished in GluA1 phosphorylation site serine-845 mutant mice. Our findings provide strong evidence that peripheral nerve injury induce long-term enhancement of cortical-spinal projecting cells in the ACC. Direct top-down projection system provides rapid and profound modulation of spinal sensory transmission, including painful information. Inhibiting cortical top-down descending facilitation may serve as a novel target for treating neuropathic pain.

## Introduction

Chronic pain is a major health problem that causes economic loss world-wide. The lack of effective drugs to control chronic pain, especially neuropathic pain, is in part due to our poor understanding of the basic neurobiology of pain at the molecular and cellular levels [1-4]. For example, what changes occur in the brain in response to peripheral insults? Are these changes long-lasting? If so, do these changes affect subsequent sensory processes after injury? Long term plasticity in synaptic transmission is believed to be the key cellular mechanism for not only learning and memory, but also for storing sensory information in the brain [5-8]. In the case of chronic pain, peripheral injury triggers long term potentiation (LTP) in the spinal dorsal horn and cortical synapses, suggesting that LTP serves as the cellular model for chronic pain [3,4,9-12].

The anterior cingulate cortex (ACC) is believed to be important for mediating emotional and attentive responses to internal and external noxious stimuli [3,4,13-16]. Various electrophysiological experiments have demonstrated that ACC neurons respond to noxious stimuli in different species including mouse, rat, rabbit, monkey and human [17-20]. More recently, works based on animal models of chronic pain have begun to reveal the cellular and molecular mechanisms of pain-induced LTP in the ACC (see [4] for review). It has been found that excitatory synaptic transmission in the layer II/III neurons of ACC could be enhanced by peripheral inflammation, nerve injury or digit amputation [21-23]. Furthermore, theta-burst stimulation (TBS) induced late-phase LTP in the ACC was occluded in animals with nerve injury [21]. In accordance with synaptic studies, inhibiting or erasing LTP in the ACC can reduce behavioral hyperalgesia [21,24,25], suggesting that they share similar neuronal mechanisms [4].

It is well known that spinal nociceptive transmission receives descending inhibitory and facilitatory modulation from supraspinal structures such as the midbrain periaqueductal grey (PAG) and rostral medical medulla (RVM) [26-31]. Although a previous study reported that stimulation of the ACC facilitated spinal tail-flick reflex by acting through brainstem descending modulation system [32], few works on the possible direct top-down corticospinal modulation in pain have been reported. Previous anatomic studies report that some prefrontal cortical areas, including part of the dorsal ACC, send descending projections to the spinal cord in rats and monkeys [33,34]. This link provides possible pathway for ACC neurons to directly regulate the spinal cord neurons. In the present study, we employ integrative experimental approaches to show that long-term plastic changes taking place in these spinal cord projecting neurons in the deep layers of the ACC after nerve injury. The potentiated corticospinal projection will play direct and potent effects in pain regulation.

## Results

### Corticospinal projections from the ACC in adult mice

**Figure 1:**
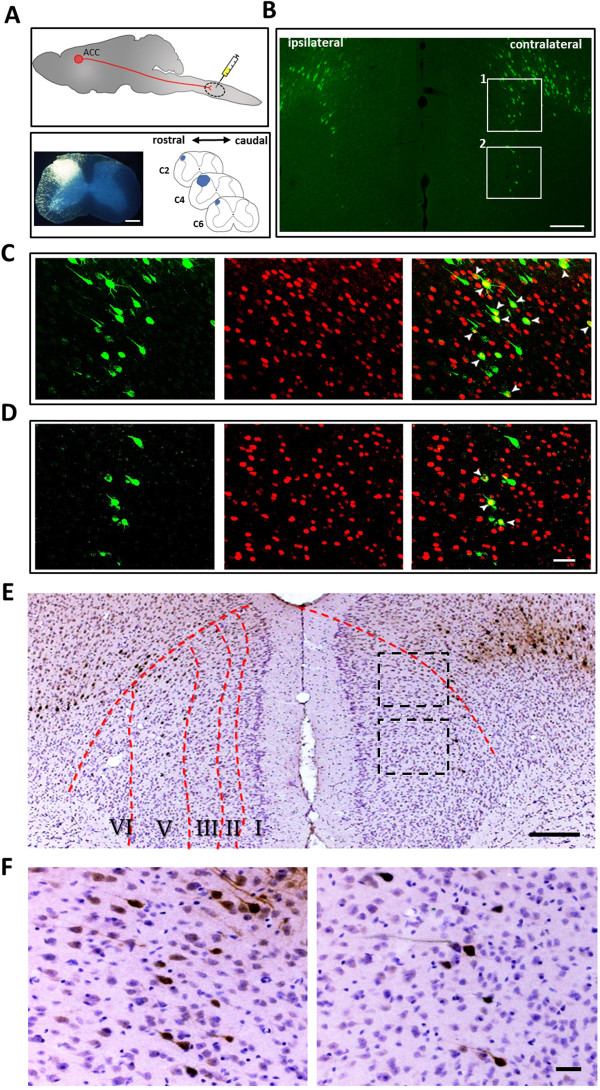
**Fos/FG double labeling after Fluoro-Gold injection into the spinal cord. A**, Schematic figures and digitized photomicrograph showing Fluoro-Gold (FG) injection site in the spinal cord and retrograde transportation of FG label neurons in the ACC. **B**, Distribution of FG labeled neurons in both sides of ACC after FG injection into the spinal cord. **C**, **D**, Augmented figures showing FG (green) and Fos (red) double-labeling results in rectangle area 1 **(C)** and 2 **(D)** in **B**. Arrowheads on the merged figures indicate FG/Fos double-labeled neurons. **E**, After FG injection into one side of the dorsal part of spinal cord, the FG retrogradely labeled cells in the ACC was immunostained with anti-FG antibody and shown with ABC method. With Nissl counterstaining, most of the FG labeled cells are shown to be located within the layer V of the ACC. **F**, Augmented figures from upper (left) and lower (right) rectangled areas in **E** showed FG labeled neurons located in the layer V of the ACC. Bars equal to 200 μm in **A**, **B** and **E** and 20 μm in **C**, **D** and **F**.

Since the dorsal horn of spinal cord (SC) is important for the transmission of nociceptive information, we firstly tested whether there were direct projections from the ACC to the spinal dorsal horn in adult mice. To investigate this, we injected the retrograde tracer Fluoro-Gold (FG) into the dorsal horn of mouse SC (n = 6 mice) (Figure 1A). Seven days after injection, FG-retrogradely labeled cortical neurons were observed in the bilateral sides of the ACC from 0.3-1.1 mm anterior to the bregma, with the contralateral predominance (71.3 ± 3.3% of FG labeled cells) (Figure 1B). Most of the FG retrograde labeled neurons were located in layer V, with scattered FG labeled neurons found in layer VI. Few or no labeled cells were found in the superficial layers (layers I-III) (Figure 1E-F). Furthermore, more FG-labeled neurons were found in the dorsal ACC (Table 1).

**Table 1:** Numbers of Fos-immunoreactive (ir), FG-labeled and Fos/FG dual-labeled neurons (dorsal/ventral) in the contralateral anterior cingulate cortex after FG injection into the spinal cord

**Mouse**	**Nerve injury**				**Sham surgery**		
	**Fos-ir neurons**	**FG-labeled neurons**	**Fos/FG dual-labeled neurons (%1; %2)**		**Fos-ir neurons**	**FG-labeled neurons**	**Fos/FG dual-labeled neurons (%1; %2)**
**M1**	933/416	178/21	121/2 (9.1; 61.8)	**M4**	103/33	221/15	19/2 (15.4; 8.9)
**M2**	781/326	226/52	200/29 (20.7; 82.4)	**M5**	112/31	146/9	17/0 (11.9; 11.0)
**M3**	1152/368	155/20	109/13 (8.0; 69.7)	**M6**	121/42	155/10	24/7 (19.0; 18.8)

The two sets of data in each bracket indicates the number of neurons located in the dorsal or the ventral part of the ACC, respectively.

%1: the percentage of total Fos/FG dual-labeled neurons (the sum of dorsal and ventral part of the ACC) to total Fos-ir neurons. %2: the percentage of total Fos/FG dual-labeled neurons to total FG-labeled neurons.

**Figure 2:**
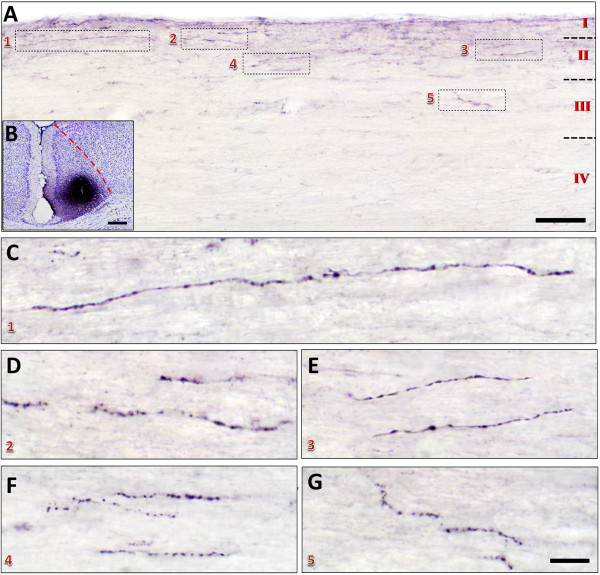
**Distribution of Pha-L anterograde labeled fibers and terminals in the spinal dorsal horn projected from the ACC. A-B**, One sample figure from a sagittal slice **(A)** showing that Pha-L labeled fibers and terminals were distributed in the laminae I-III of the spinal cord (c4) after Pha-L injection into one side of the ACC **(B)**. The rectangled areas **(1–5)** in **A** were augmented in **C-G** respectively. Bars equal to 100 μm in **A**, 200 μm in **B**, 10 μm in **C-G**.

**Figure 3:**
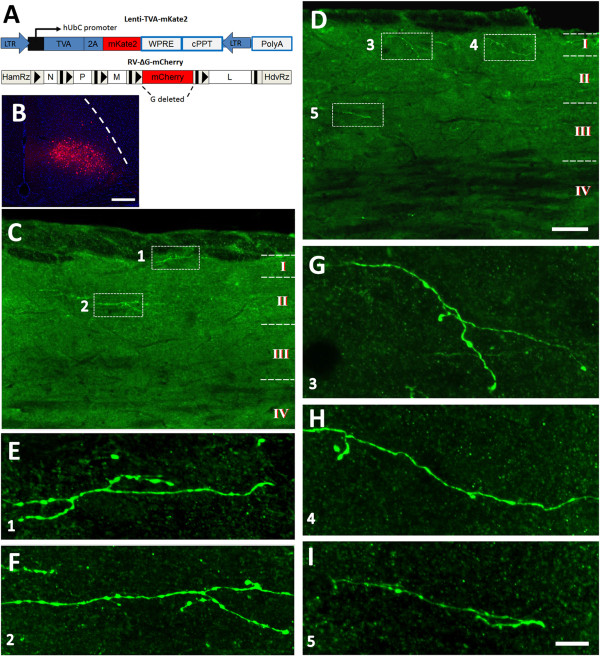
**Distribution of rabies virus anterograde labeled fibers and terminals in the spinal dorsal horn projected from the ACC. A**, The schematic figure showing the design of lentivirus-assistant rabies virus used for anterograde tracing. **B-D**, After the virus injection into one side of the ACC **(B)**, virus infected fibers and terminals were distributed in the laminae I-III of the spinal cord (c4) **(C-D)**. Rectangled areas of **1**–**5** in **C** and **D** were augmented in **E-I** respectively. Bars equal to 200 μm in **B**, 50 μm in **C** and **D**, 10 μm in **E-I**.

Injection of FG may label passing nerve fibers near the injection area. We thus explored anterograde labeling methods to further confirm the direct projections from the ACC to the spinal dorsal horn. We firstly injected phaseolus vulgaris leucoagglutinin (Pha-L), a widely used anterograde neuronal tracer [35,36], into the ACC. Two weeks later, the Pha-L anterograde labeled fibers and terminals were detected in the dorsal layers of the spinal cord, in which most of the varicose and punctate fibers and terminals were distributed in laminae I and II and scattered fibers and terminals were observed in lamina III. No obvious fibers and terminals could be observed in deeper layers (Figure 2). Traditional anterograde tracers, including the Pha-L may have diffusion capacity in injected sites [37], we next used a modified anterograde tracing strategy based on lentivirus-assistant rabies virus system to only stain a small region of the deep ACC and check their projections to the spinal cord. The vesicular stomatitis virus glycoprotein (VSV-G) pseudotyped Lenti-TVA-mKate infected the neurons anterogradely, which located in the ACC and expressed the avian receptor protein (TVA) and mKate restrictlly in the infected neurons (Figure 3) [38]. The rabies virus EnvA-RV-mcherry was a glycoprotein deleted virus and was pseudotyped with the avian sarcoma leucosis virus glycoprotein (EnvA) [39,40]. The EnvA-RV-mcherry could only infect the neurons that express TVA and labeled these Lenti-TVA-mKate infected ACC neurons locally (Figure 3A). We found that one week after the rabies infection in limited group of neurons in the deep layers of ACC (Figure 3B), virus infected varicose fibers and terminals (immunostained with FITC) were detected in the superficial layers (laminae I-III) of the spinal cord. Although the number of virus infected fibers and terminals was significantly less than that of Pha-L labeled ones, their distribution patterns were similar (Figures 2 and 3).

### Peripheral nerve injury increased Fos expression in spinal cord but not ventral striatum projecting neurons in the layer V of the ACC

**Figure 4:**
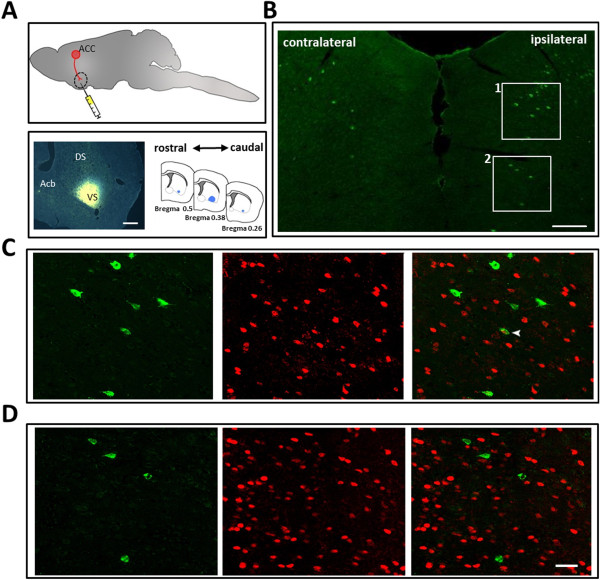
**Fos/FG double labeling after Fluoro-Gold injection into the ventral striatum. A**, Schematic figures and digitized photomicrograph showing FG injection site in the ventral striatum and retrograde transportation of FG to label neurons in the ACC. **B**, Distribution of FG labeled neurons in both sides of ACC with FG injection into the ventral striatum. **C-D**, Augmented figures showing FG (green) and Fos (red) double-labeling results in rectangle area 1 **(C)** and 2 **(D)** in **B**. Arrowheads on the merged figures indicate FG/Fos double-labeled neurons. Bars equal to 1000 μm in **A**, 200 μm in **B**, and 20 μm in **C** and **D**.

Confirmation of the projections from the ACC to the SC leads us to wonder if they are related to pain regulation. We then tested the expression of Fos protein, a widely used activity marker [41], in mice exposed to common peroneal nerve (CPN) ligation surgery – a model of neuropathic pain [21]. As expected, significantly more expression of Fos protein was observed in layer V neurons of the ACC in mice with nerve injury as compared with mice receiving sham surgery (Table 1). Among ACC- SC projecting neurons, many of them expressed Fos after nerve injury (mean 71.3 ± 7.3%) (n = 3 mice) (Figure 1C-D) (Table 1). In comparison, we tested the Fos expression in the ACC-ventral striatum (VS) projecting neurons (Figure 4A), which are more likely to be involved in reward function [42]. After FG injection into the VS, FG labeled neurons were observed in the bilateral ACC (Figure 4B). Unlike the corticospinal projecting cells, most of the ACC-VS projecting neurons were found in the ipsilateral ACC (Table 2). FG labeled neurons were distributed mainly in layer V of the dorsal part of the ACC, with scattered neurons in the layers III and VI, but no detectable FG labeled cells in layers I and II. Moreover, Fos staining revealed only small percentage of ACC-VS projection cells were activated after nerve injury (Table 2; Figure 4C-D).

**Table 2:** Numbers of Fos-immunoreactive (ir), FG-labeled and Fos/FG dual-labeled neurons (dorsal/ventral) in the ipsilateral anterior cingulate cortex after FG injection into the ventral striatum

**Mouse**	**Nerve injury**				**Sham surgery**		
	**Fos-ir neurons**	**FG-labeled neurons**	**Fos/FG dual-labeled neurons (%1; %2)**		**Fos-ir neurons**	**FG-labeled neurons**	**Fos/FG dual-labeled neurons (%1; %2)**
**M1**	845/310	116/12	9/2 (1.0; 8.6)	**M4**	85/24	120/11	10/1 (10.1; 8.4)
**M2**	779/298	101/16	12/3 (1.4; 12.8)	**M5**	106/68	132/15	15/3 (10.3; 12.2)
**M3**	959/294	116/11	15/5 (1.6; 15.7)	**M6**	96/35	106/10	11/4 (11.5; 12.9)

The two sets of data in each bracket indicates the number of neurons located in the dorsal or the ventral part of the ACC, respectively.

%1: the percentage of total Fos/FG dual-labeled neurons (the sum of dorsal and ventral part of the ACC) to total Fos-ir neurons. %2: the percentage of total Fos/FG dual-labeled neurons to total FG-labeled neurons.

### Potentiated AMPA receptor (AMPAR)-mediated postsynaptic responses

**Figure 5:**
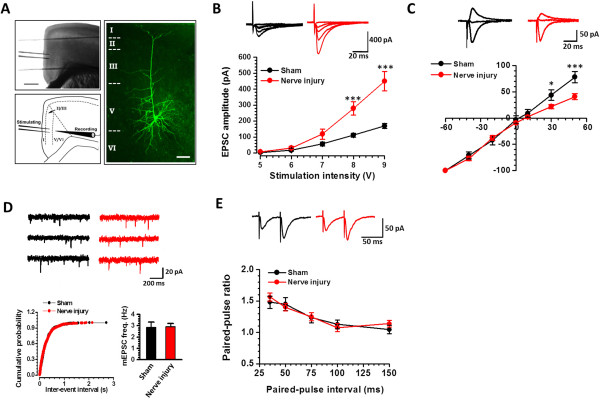
**Nerve injury increased the AMPAR mediated postsynaptic responses in the ACC. A**, Schematic figures showing the recording on layer V by stimulating shallow layers (II/III), plus one biocytin labeled layer V pyramidal cell. **B**, The input–output curve of ACC layer V neurons became steeper in mice with nerve injury, compared with mice with sham injury. Two-way ANOVA followed by Tukey’s *post hoc* test; **C**, An inward rectification of AMPA I-V curve of layer V neurons after nerve injury. **D-E**, The frequency of the mEPSC **(D)** and the paired-pulse ratio **(E)** is not changed after nerve injury.

Previous studies in the ACC found that excitatory transmission in layer II-III pyramidal cells are potentiation after peripheral nerve injury or inflammation (see [3,4] for reviews). Little information is available about excitatory synaptic transmission in deep cingulate neurons. Therefore, we decided to record the AMPAR mediated excitatory postsynaptic currents (EPSCs) on layer V pyramidal cells in the ACC to explore whether their synaptic responses are also enhanced after nerve injury (Figure 5A). We found that the input (stimulation intensity)–output (EPSC amplitude) curve (I-O curve) of AMPAR responses had steeper slope after peripheral nerve injury, compared with that of neurons from the sham surgery group (n = 12 neurons/9 mice in each group, two way ANOVA followed with Tukey’s post hoc test, *F*_*(1, 110)*_ = 42.147, *p* < 0.001) (Figure 5B), indicating that excitatory responses are potentiated after nerve injury. The AMPAR mediated EPSCs at the different holding potentials (-60 to +50 mV) were also recorded, and we found an obvious inward rectification of the mean *I-V* curve in mice with nerve injury (sham surgery: n = 7 neurons/6 mice, nerve injury: n = 9 neurons/7 mice; *p* < 0.05) (Figure 5C).

To determine if presynaptic transmitter release is altered in response to nerve injury, we measured the frequency of the miniature EPSCs (mEPSC) and the ratio of paired-pulse facilitation (PPF), two simple measurements for presynaptic transmitter release possibility. After nerve injury, no change in the frequency of the mEPSCs (n = 18 neurons/9 mice in each group, *p* > 0.05) and PPF ratios (n = 18 neurons/9 mice in each group, two way ANOVA, *F*_*(1, 170)*_ = 0.161, *p* > 0.05) were detected (Figure 5D-E). These results suggest that presynaptic release of glutamate is unlikely enhanced on layer V pyramidal cells by nerve injury.

**Figure 6:**
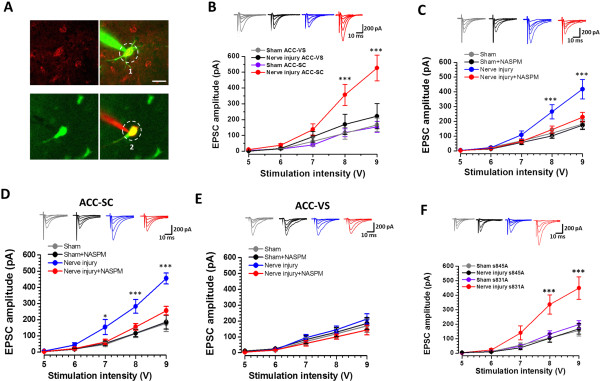
**GluA1/3 mediated the potentiated input–output responses in ACC-spinal cord projecting neurons. A**, Digitized photomicrograph showing one DiI retrogradely labeled neuron after injection into the spinal cord (SC) was whole-cell patched and dual labeled by intracellular injection of Alex-488 (1), and one Alex-488 Dextran retrogradely labeled neuron after injection into the ventral striatum (VS) was patched and dual labeled with Alex-594(2). **B**, Samples and summarized results showing the I-O curve in ACC-SC projecting neurons in mice with nerve injury has steeper slope, as compared with ACC-SC projecting neurons in mice with sham surgery. Meanwhile, the I-O curve in ACC-VS projecting neurons was not different in mice with or without nerve injury. **C**, Bath application of NASPM only inhibited the I-O responses of neurons from nerve injury but not sham surgery group. **D**, Bath application of NASPM only inhibited the I-O responses of ACC-SC projecting neurons from nerve injury but not sham surgery group. **E**, Bath application of NASPM inhibited the I-O responses of ACC-VS projecting neurons from neither nerve injury nor sham surgery group. **F**. Nerve injury enhanced the I-O responses of ACC-SC projecting neurons in s831A mice but not in s845A mice. Bar equals to 20 μM in **A**. *,*p* < 0.05; ***,*p* < 0.001.

Fos staining works suggest that SC but not VS projecting neurons in the ACC are more likely to be activated after nerve injury. It is important to determine if AMPAR mediated EPSCs are selectively enhanced in ACC-SC projecting neurons. After retrograde labeling ACC projecting cells by DiI (0.25%) or Alexa-488 conjugated Dextran (10%) (Figure 6A), we performed electrophysiological recordings from retrogradely labeled cells that were randomly selected from both sides of the ACC. We found that the I-O curve of AMPAR mediated EPSCs of spinal cord projecting neurons has steeper slope in mice with nerve injury, as compared with SC projecting neurons of mice with sham surgery (sham surgery: n = 6 neurons/5 mice. nerve injury: n = 12 neurons/9 mice; Two-way ANOVA followed with Tukey’s post hoc test, *F*_*(1, 80)*_ = 22.461, *p* < 0.001). Interestingly, nerve injury did not affect the I-O curve in ACC-VS projecting neurons (sham surgery: n = 6 neurons/5 mice. nerve injury: n = 8 neurons/6 mice; Two way ANOVA, *F*_*(1, 60)*_ =1.531, *p* > 0.05) (Figure 6B).

### Calcium-permeable AMPAR (CP-AMPAR) contributes to the potentiation

**Figure 7:**
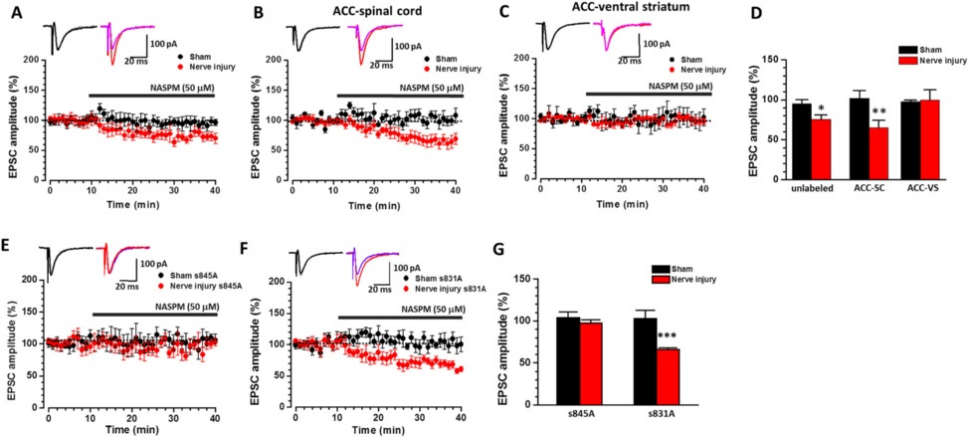
**GluA1/3 mediated the potentiation of the AMPAR mediated basal synaptic transmission in ACC-spinal cord projecting neurons. A**, Samples and the averaged results showing that NASPM inhibited the AMPAR EPSCs in mice with nerve injury but not in mice with sham surgery. **B**, Samples and averaged results showing that NASPM only inhibited the AMPAR EPSCs in ACC-SC projecting neurons in mice with nerve injury but not in mice with sham surgery. **C**, NASPM did not inhibit the AMPAR EPSCs in ACC-VS projecting neurons in mice either with or without nerve injury. **D**, Plotted figure shows the summarized effect of NASPM on unlabeled or retrograded labeled projecting neurons. **E-G**, NASPM can only inhibit the AMPAR EPSCs of ACC-SC projecting neurons in s831A mice with nerve injury, but not in s845A mice with nerve injury, as well as s831A and s845A mice with sham surgery. *,*p* < 0.05; **,*p* < 0.01; ***,*p* < 0.001.

AMPAR is heterotetramer of four homologous subunits (GluA1 to GluA4) that combine in different stoichiometries to form different subunits [43]. In normal conditions, most of the AMPAR contain the GluA2 subunit. During synaptic plastic changes, GluA2 can be replaced by GluA1/3 subunit [44,45], which is Ca^2+^ permeable AMPAR (CP-AMPAR) and inwardly rectifying [46]. According to the observed inward rectification of the mean *I-V* curve in mice with nerve injury (Figure 5C), we expect that the potentiated AMPAR mediated responses in the ACC layer V may be sensitive to the inhibition of CP-AMPAR antagonist NASPM. We next recorded AMPAR mediated responses from ACC neurons in mice with nerve injury and found that NASPM inhibited the I-O responses (*F*_*(1, 80)*_ = 29.163, *p* < 0.001, n = 9 neurons/6 mice, two way ANOVA followed with Tukey’s *post hoc* test). Meanwhile, NASPM didn’t inhibit the I-O responses in mice with sham surgery (*F*_*(1, 80)*_ =0.849, *p* > 0.05, n = 9 neurons/6 mice, two way ANOVA) (Figure 6C). Furthermore, the AMPAR mediated eEPSCs were significantly inhibited by bath application of NASPM (50 μM) (75.3 ± 6.0% of baseline; n = 8 neurons/7 mice, paired *t*-test, *p* < 0.05). The same application of NASPM did not affect AMPAR mediated responses in ACC neurons recorded from mice with sham surgery (95.0 ± 5.2% of baseline; n = 7 neurons/6 mice, *p* > 0.05) (Figure 7A).

ACC-SC but not ACC-VS projecting neurons showed increased Fos expression, as well as potentiated AMPAR mediated responses. We therefore further investigated the effect of NASPM on these projecting neurons. After bath application of NASPM, the enhanced I-O responses on ACC-SC projecting neurons in mice with nerve injury (*F*_*(1, 70)*_ =24.576, *p* < 0.001, n = 8 neurons/5 mice, two way ANOVA followed with Tukey’s *post hoc* test) were inhibited. This inhibition was not observed on ACC-SC projecting neurons in mice with sham surgery (two way ANOVA, *F*_*(1, 60)*_ =0.052, *p* > 0.05, n = 7 neurons/5 mice) or on ACC-VS neurons in mice with either sham surgery (*F*_*(1, 60)*_ =0.402, *p* > 0.05, n = 7 neurons/6 mice, two way ANOVA) or nerve injury (*F*_*(1, 60)*_ =1.320, *p* > 0.05, n = 7 neurons/5 mice, two way ANOVA) (Figure 6D, E). In consistent with this finding, AMPAR mediated eEPSCs were greatly inhibited in ACC-SC projecting neurons in mice with nerve injury (65.7 ± 7.7% of baseline; n = 8 neurons/7 mice, paired *t*-test, *p* < 0.01) but not in mice with sham treatment (101.9 ± 7.8% of baseline; n = 6 neurons/5 mice, paired *t*-test, *p* > 0.05) (Figure 7B, D). Furthermore, AMPAR mediated eEPSCs from ACC-VS projecting neurons were not affected by bath application of NASPM (sham surgery: 103.6 ± 11.4% of baseline, n = 6 neurons/5 mice; nerve injury, 98.4 ± 1.9% of baseline, n = 7 neurons/5 mice. paired *t*-test, *p* > 0.05) (Figure 7C-D).

### GluA1 PKA phosphorylation site is important for nerve injury induced synaptic potentiation

Phosphorylation of GluA1 is important for GluA1 trafficking and synaptic plasticity [47,48]. Previous studies showed that nerve injury increased GluA1 PKA phosphorylation at the serine 845 site in the ACC, by using western blot method [22]. However, it is unknown if PKA phosphorylation of GluA1 is required for nerve injury induced synaptic potentiation of ACC neurons. Taking advantage of genetically induced GluA1 phosphorylation site knock in mice [45], we performed electrophysiological recordings from ACC-SC projecting neurons to test if these mutations affected injury induced synaptic potentiation. We found that enhanced AMPAR I-O responses after the injury was completely abolished in PKA phosphorylation s845A mutant mice (sham surgery: n = 7 neurons/6 mice; nerve injury: n = 7 neurons/5 mice, Two way ANOVA, *F*_*(1, 60)*_ = 0.028, *p* > 0.05), but not in PKC phosphorylation s831A mice (sham surgery: n = 7 neurons/6 mice; nerve injury: n = 8 neurons/7 mice, Two way ANOVA followed with Tukey’s post hoc test, *F*_*(1, 65)*_ =22.339, *p* < 0.001) (Figure 6F). Baseline AMPAR responses were not different in these two lines of mice. We further examined the effect of NASPM on the ACC-SC projecting neurons in s845A and s831A mice. We found that bath application of NASPM significantly inhibited AMPAR eEPSCs in PKC phosphorylation s831A mice (65.8 ± 2.0% of baseline; n = 7 neurons/6 mice, paired *t*-test, *p* < 0.001) but not in PKA phosphorylation s845A mice (97.6 ± 3.6% of baseline; n = 6 neurons/6 mice, paired *t*-test, *p* > 0.05) with nerve injury (Figure 7E-G). These results strongly suggest that CP-AMPAR accumulation in the synaptic region is prevented by the GluA1 mutation on the PKA phosphorylation site, but not on the PKC phosphorylation site.

### Excitatory unitary transmission from ACC layer III to layer V

**Figure 8:**
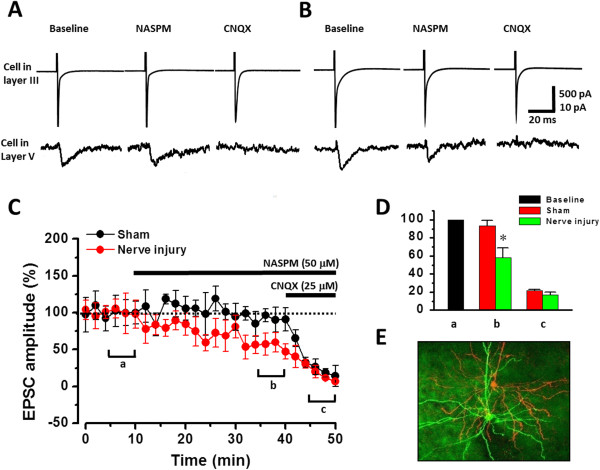
**Nerve injury enhanced the layer III-layer V unitary AMPAR responses. A-B**, Sample traces showing that NASPM inhibit the layer III-layer V unitary AMPAR responses in mice with nerve injury but not in mice with sham surgery. **C-D**, Summarized data showing the effect of NASPM on the layer III-layer V unitary AMPAR responses in mice with nerve injury or sham surgery. AMPA/KA receptor antagonist CNQX blocked the AMPA current. **E**, Fluorescent figure showing one pair of neurons labeled with biocytin (stained with FITC, green, layer V) or Lucifer yellow (red, layer III). *,*p* < 0.05.

It has been proposed that ACC superficial layer (II/III) cells send their projections to deeper layer V/VI cells [49,50]. To determine if inter-layer excitatory synapses may undergo potentiation after injury, we performed dual patch recordings in ACC neurons of mice aged 6–7 weeks. In a total of 14 mice (88 dual recording experiments), three pairs of neurons between presynaptic layer III and postsynaptic layer V connections were obtained from sham surgery and nerve injury groups, respectively. Action potentials (APs) were induced in presynaptic (layer III) neurons by a brief (1 ms) depolarizing voltage pulse (from -60 mV to +20 mV) at 0.05 Hz and postsynaptic unitary AMPAR EPSCs were thus recorded [51]. We found that presynaptic AP-evoked postsynaptic EPSCs were inhibited by bath applications of NASPM (50 μM) in ACCs from mice with nerve injury (58.3 ± 10.8% of baseline, paired *t*-test, *p* < 0.05) but not from mice with sham surgery (93.4 ± 5.6% of baseline, paired *t*-test, *p* > 0.05) (Figure 8). These findings suggest that injury induced plastic changes take place in local excitatory synapses, especially those linking layer III to layer V ACC neurons.

### Electrophysiological recordings from injury activated Fos-positive ACC layer V neurons

**Figure 9:**
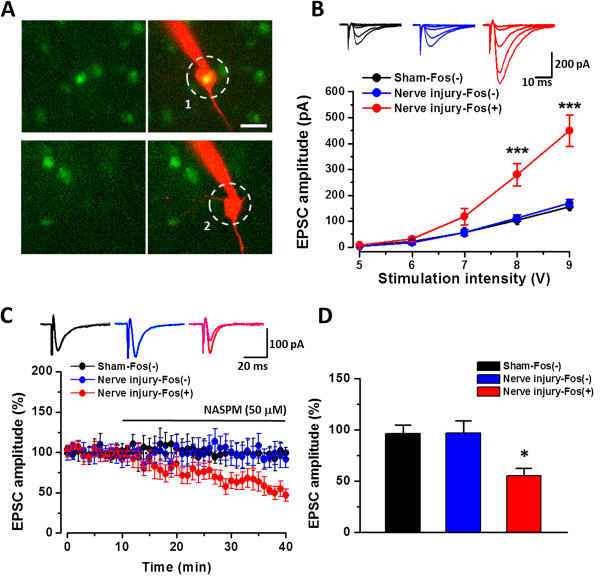
**GluA1/3 mediated the potentiated AMPAR current on the Fos-positive layer V neurons in mice with nerve injury. A**, Digitized photomicrograph showing one Fos-GFP-positive (Fos+) (1) or one Fos-GFP-negative (Fos-) (2) neuron was whole-cell patched and dual-labeled by intracellular injection of Alex-594. **B**, Samples and summarized results showing the AMPAR I-O curve recorded on Fos- neurons in mice with sham surgery (Sham-Fos-) and on Fos- (Nerve injury-Fos-) and Fos + (Nerve injury-Fos+) neurons in mice with nerve injury, respectively. **C**, Samples and averaged results showing that NASPM only inhibited the AMPAR EPSCs on Fos + neurons in mice with nerve injury but not on Fos- neurons in mice either with nerve injury or sham surgery. **D**, Plotted figure showing the summarized effect of NASPM on Sham-Fos-, Nerve injury-Fos- and Nerve injury-Fos + neurons. Bar equals to 20 μm in **A**. *, *p* < 0.05; ***, *p* < 0.001.

To test whether AMPAR mediated currents are enhanced in injury triggered Fos-positive layer V neurons, we used transgenic mice in which the expression of GFP is controlled by the promoter of the *c-fos* gene [52,53]. After peripheral injury, the *c-fos* gene was activated and neurons can therefore be detected with GFP expression in transgenic mice [21]. Seven days after nerve injury, strong FosGFP-positive neurons were found in layer V of the ACC in Fos-GFP mice (Figure 9A). We then performed whole-cell patch recording from FosGFP-positive (Fos+) neurons. Recordings from FosGFP-negative (Fos-) neurons were also performed in the same slices for the comparison. We found that the I-O curves of AMPAR EPSCs significantly shifted to the left in Fos + neurons of mice with nerve injury, compared with those from Fos- neurons in mice with nerve injury or Fos- neurons in sham-operated mice (sham surgery: n = 9 Fos- neurons/6 mice, nerve injury: n = 9 Fos- neurons/6 mice and 10 Fos + neurons/7 mice, Two way ANOVA, *F*_*(2, 139)*_ = 25.293, *p* < 0.001) (Figure 9B). We then applied NASPM to Fos+ or Fos- neurons in mice with nerve injury or Fos- neurons in mice with sham surgery. In mice with nerve injury, the AMPAR mediated eEPSCs of Fos + neurons were inhibited significantly (n = 7 neurons/7 mice, 55.1 ± 7.6% of baseline; paired *t*-test, *p* < 0.05) but not in Fos- neurons (n = 6 neurons/6 mice, 97.0 ± 11.8% of baseline; *p* > 0.05). Moreover, AMPAR mediated responses on Fos- neurons from sham surgery mice were not affected either (n = 5 neurons/5 mice, 96.3 ± 8.3% of baseline; *p* > 0.05) (Figure 9C-D).

## Discussion

In the present study, we have demonstrated that postsynaptic recruitment of GluA1 mediated the nerve injury induced LTP, especially on the corticospinal projecting neurons of the ACC in adult mice. To our knowledge, this is the first study showing that nerve injury induces LTP in pain activated cortical–spinal cord projecting neurons in the deep layers of the ACC. Potentiation is mediated by postsynaptic AMPARs, and cAMP-PKA dependent pathway plays a critical role in this LTP. These findings provide strong evidence for the first time that potentiated responses in deep cingulate neurons may subsequently enhance or facilitate spinal pain transmission by direct cortical-spinal projecting control. This new mechanism allows synaptic potentiation at single synapse level (i.e., postsynaptic sites of deep cingulate pyramidal cells) to influence sensory pain transmission at distal location, at dorsal horn of the spinal cord. Synaptic potentiation and top-down facilitation thus may play important roles in behavioral hypersensitive responses to sensory stimuli in chronic pain conditions. Our data provide novel evidence for the corticospinal pathway as a target for reducing chronic pain.

### Direct corticospinal projections from the ACC

Previous studies from rats and monkeys show that some of ACC neurons send their projecting fibers to the spinal cord [33,34]. In the present study, by using different anatomic methods, we have clearly demonstrated that deep ACC neurons send direct descending projecting fibers to the spinal cord, especially the dorsal horn for the spinal cord in adult mice. This top-down corticospinal projection system is likely important for functional pain modulation, since our previous study using in vivo preparation show that spinal nociceptive tail-flick reflex is facilitated by activation of the ACC [32]. Although our previous studies found that descending modulation of the tail-flick reflex depends on brainstem relay, we cannot rule out that top-down descending facilitation modulation may not require brainstem relay in certain conditions. Direct descending projecting pathways could provide fast modulation of spinal synaptic transmission in an efficient manner. Our preliminary electrophysiological studies using *in vivo* whole-cell patch-clamp recording technique found that ACC stimulation indeed facilitated excitatory glutamatergic transmission in the spinal cord dorsal horn of adult rats (unpublished data).

### Potentiation of excitatory transmission after nerve injury

Recent studies have consistently shown that excitatory transmission in the layer II/III of the ACC is potentiated after peripheral nerve injury (see [4]). Both presynaptic and postsynaptic mechanisms contribute to the potentiation [22]. The present results show that excitatory synaptic transmission in the layer V/VI of the ACC is also potentiated. However, unlike layer II/III, potentiation of excitatory transmission in layer V/VI is mainly mediated by postsynaptic mechanisms. However, we cannot rule out the possibility that some presynaptic mechanism may also contribute to this potentiation. Similar to layer II/III, calcium-CaM dependent PKA signaling pathway is critical for potentiation in layer V/VI neurons. By using dual paired recordings, we found that inter-cortical connections between layer II/III cells and layer V cells are also potentiated.

### ACC LTP and postsynaptic GluA1

GluA1 trafficking into the synaptic region is an important mechanism for postsynaptic form of LTP [43,47,54,55]. Our previous studies using pharmacological and genetic approaches consistently demonstrate that GluA1 is critical for ACC LTP [3,56,57]. In the present study, by applying GluA1/3 antagonist NASPM, we confirm that enhanced postsynaptic GluA1/3 may contribute to the LTP in ACC-SC projecting neurons after nerve injury. In a previous study, we observed through western blot analysis that nerve injury increases phosphorylated-GluA1 expression in layer II/III neuronal membrane in the ACC [22]. However, it is unknown if this PKA phosphorylation is actually required for chronic pain induced LTP. Through the use of mice with PKC or PKA phosphorylation site mutations, we showed that PKA phosphorylated site ser-845 but not PKC phosphorylated site ser-831 on GluA1 is necessary for the potentiated AMPAR mediated responses in ACC-SC projecting neurons. This finding is consistent with previous work in the ACC that show the requirement of AC1-cAMP signaling pathway for the induction of ACC LTP. ACC LTP is blocked in gene knockout mice lacking AC1 [58], and AC1 inhibitor NB001 prevented the induction of LTP [24].

### Top-down descending facilitation

Spinal nociceptive transmission is under biphasic modulation from supraspinal structures, especially descending facilitatory modulation [31,59]. Most of previous studies have mainly focused on descending projections from brainstem neurons [2,27,60,61], and few studies have reported synaptic plastic changes in these cortical-spinal top-down projection cells. Using behavioral nociceptive reflexes, we have previously shown that ACC stimulation induces the facilitation of the spinal nociceptive tail-flick reflex [32]. Pharmacological studies revealed that some of this descending facilitation may rely on brainstem RVM cells, and the spinal transmitter serotonin is likely a key mediator for such facilitation [62,63]. In the present study, we reveal a direct cortical-spinal projecting pathway. Our preliminary studies show that ACC-spinal cord descending facilitation does not require the brainstem relay (Chen et al., unpublished data). Our *in vivo* electrophysiological studies show that ACC stimulation can facilitate spinal cord neurons in control condition, while this facilitation is blocked in chronic pain condition, suggesting that ACC-SC descending facilitation is tonically activated in neuropathic pain condition (Chen et al., unpublished data). Considering glutamate is the major transmitter for most of pyramidal cells in the ACC, it raises the possibility that glutamate may also act as a transmitter for facilitating pain transmission in the spinal cord. Different types of glutamate receptors in the spinal cord, such as NMDA receptor, kainate receptors, metabotropic glutamate receptors as well as the possible recruitment of postsynaptic AMPARs may acts as possible candidates mechanisms for the amplified excitation in spinal cord [64-66]. Future studies are clearly needed to reveal molecular mechanism for this novel modulation.

### Functional implications

**Figure 10:**
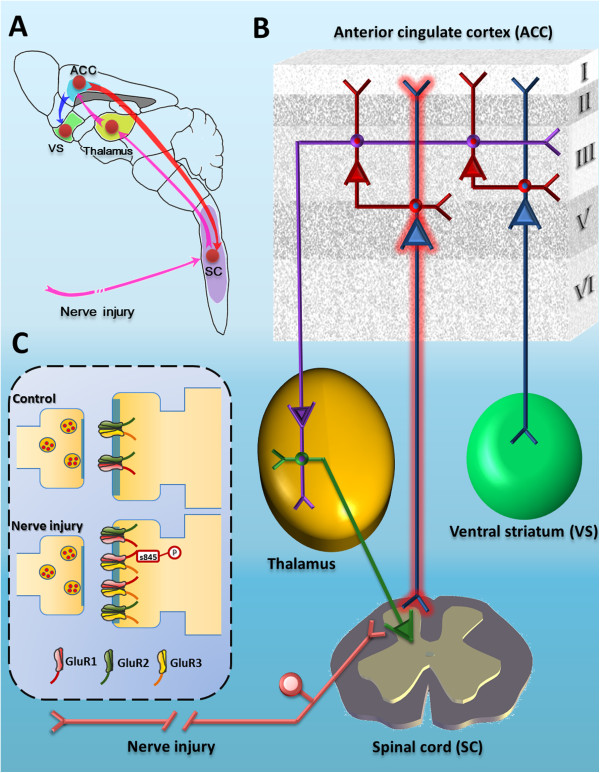
**A model for the role of cingulate-spinal projection pathways in pain regulation and their synaptic plasticity after nerve injury. A**, A diagram shows the spinal-cortex-spinal circuit containing the spinal cord dorsal horn and ACC in the transmission and modulation of sensory information in chronic pain conditions. Through the thalamus (Thal), nociceptive information reaches the neurons in the ACC from the spinal cord dorsal horn. These information affect neural activities of deeper cingulate neurons by direct thalamic projecting or intercortical inputs from layer II and III cells. Many of deep cingulate neurons then send their direct projections to the spinal cord, and possibly affect spinal pain transmission. **B**, Detailed cortical circuits within the ACC. Pyramidal cells in layer II/III form direct synaptic connections with neurons in layer V/VI within the ACC. Enhanced synaptic transmission from Layer II/III to V is likely to activate or enhance descending facilitatory modulation from the ACC to the spinal cord. **C**, A synaptic model shows postsynaptic potentiation of excitatory transmission in layer V cells after peripheral nerve injury. PKA-dependent AMPARs GluR1/3 subtypes insertion is likely a key cellular mechanism for this postsynaptic potentiation.

The present study provides strong evidence for positive feedback mechanism at both synaptic and circuit levels in chronic pain conditions (see Figure 10). At the synaptic level, this is the first study to show that AMPARs undergo up-regulation in corticospinal projecting cells from the ACC. Accordingly, we presented pharmacological and genetic studies confirming the necessity of GluA1 receptors in ACC potentiation. At circuit level, our results suggest that potentiated ACC synapses may enhance neuronal spike responses to incoming sensory inputs from the thalamus. Consequently, the firing of corticospinal ACC neurons may trigger spinal facilitation of sensory transmission, including painful information. In case of nerve injury, this ACC-SC loop is activated and contributes to the maintenance of behavioral hyperalgesia and allodynia. These findings provide insights for designing new treatment methods and protocols, as well as exploring possible novel targets for analgesic drugs. One may reduce chronic pain by inhibiting injury triggered potentiation in the cortex, and/or inhibiting descending facilitation by corticospinal projections from the ACC. Future studies are clearly needed to identify the transmitters and mechanisms for such descending facilitation in different chronic pain conditions.

## Methods

### Animals

Adult male C57BL/6, GluA1 serine-831 and serine-845 phosphorylation site mutant (s831A and s845A) mice were used. Animals were randomly housed under a 12-h light–dark cycle (9 a.m. to 9 p.m. light), with food and water freely available, at least one week before carrying out experiments. All procedures involving animals were under the guidelines of the Fourth Military Medical University, Xi’an Jiaotong University, Wuhan Institute of Physics and Mathematics, the Chinese Academy of Sciences, University of Toronto, National Institute for Physiological Sciences and Johns Hopkins University.

### Nerve injury model

A model of neuropathic pain was induced by the ligation of the common peroneal nerve (CPN) as described previously [21,22]. Briefly, mice were anesthetized by an intraperitoneal injection of a mixture saline of ketamine (0.16 mg/kg) and xylazine (0.01 mg/kg). The CPN was visible between the anterior and posterior groups of muscles, running almost transversely. The left CPN was slowly ligated with chromic gut suture 5–0 until contraction of the dorsiflexor of the foot was visible as twitching of the digits. The skin was then sutured and cleaned. Sham surgery was conducted in the same manner, but the nerve was not ligated. The mice were used for behavior and/or electrophysiological studies on postsurgical days 7.

### Retrograde labeling

The procedure for retrograde tracer injection into the spinal cord (in the same time with CPN ligation or sham surgery) or ventral striatum (4 days after CPN ligation or sham surgery) was according to our previous works [67,68]. The anesthetic mice were fixed on a stereotaxic frame. For the spinal cord injection, the skin between scapulas was incised and paravertebral muscles were cut off and vertebral plate of the fourth cervical vertebra was exposed. The vertebral plate was removed and the intumescentia cervialis was exposed. Then 4% FG (For FG immunostaining), 0.25% DiI or 10% Alexa-488 Dextran (for whole cell patch recording) distilled in saline solution was unilaterally pressure-injected (0.1 μl) into the C4-5 spinal cord with a Hamilton microsyringe attached with a glass micropipette (tip outer diameters ranged from 10–20 μm). Those mice were allowed to survive for one week before continuous immunostaining or whole cell patch experimental procedures. For ventral striatum (VS) injection, the skull was exposed, and a hole was drilled through the skull over the VS (0.38 mm anterior to bregma, 2.0 mm lateral to the midline and 4.5 mm ventral to the surface of the skull for the VS). 4% FG, 0.25% DiI or 10% Alexa-488 Dextran was unilaterally and iontophoretically injected (3-5 μA pulsed, 7 sec on/off) for 25 min. Those mice were allowed for three days survive before immunostaining or whole cell patch experimental procedures.

### Anterograde labeling for Pha-L

The procedures for anterograde tracer phaseolus vulgaris leucoagglutinin (Pha-L; Vector Laboratories, Burlingame, CA) injection were essentially the same as described by our group previously [69]. Briefly, The anesthetic mouse was fixed on a stereotaxic frame and Pha-L was iontophoretically injected into unilateral deep layers of the ACC according to the atlas of the mouse brain (0.98 mm anterior to Bregma, 0.35 mm lateral to the midline and 1.8 mm deep from cerebral surface). Pha-L was dissolved in a mixture of 0.05 M Tris–HCl buffer and 0.5 M KCl (pH 7.6) to a final concentration of 2.5% (W/V). The driving current (positive, 3–5 μA, 7 s on/off) was delivered for 25 min. After injection, the surgical wounds were carefully sutured. Mice were allowed to survive for approximately 2 weeks before perfusion. To examine the Pha-L injection site and the distribution of anterogradely Pha-L-labeled fibers and terminals in the spinal cord, coronal sections containing ACC and sagittal sections containing spinal cord of cervical enlargement were incubated overnight with primary antibody goat-anti-Pha-L (1:500, Vector Laboratories, Burlingame, CA) in the 5 mM sodium phosphate (pH 7.4)-buffered 0.9% saline (PBS) containing 0.3% Triton X-100, 0.12% lambda-carrageenan, 0.02% sodium azide and 1% donkey serum. On the following day, the sections were incubated in the same dilution solution containing biotinylated anti-goat IgG (Vector Laboratories, 1:200) for 4 hours. They were then incubated in an ABC complex (Vector Laboratories, 1:200) for another 90 min. Subsequently, the sections were treated with 50 mM Tris–HCl buffer (pH 7.5) solution that containing 0.02% diaminobenzidine (DAB), 0.015% H_2_O_2_ and 0.04% NiCl_2_ to intensify DAB-based reaction for 5–15 min. After the reaction, the sections were mounted onto gelatin-coated glass slides, dehydrated and coverslipped. To better reveal the site of ACC, sections containing injection site were further counterstained with Nissl staining. Sections were observed under a light microscope (AH-3; Olympus, Tokyo, Japan).

### Anterograde labeling for lentivirus-assistant rabies virus

#### Virus preparation

To assist the rabies virus–mediated specific labeling of ACC neurons, a lentivirus plasmid expressing TVA and mkate2 was constructed by sub-clone the fusion fragment TVA:2A:mkate2 into the plasmid FUGW (Addgene 14883). This plasmids Lenti-TVA-mKate was packaged in 293-T cells by co-transfection with pMDL g/p RRE and pMD2.G. At 48 and 72 hours post transfection, the supernatant was collected and concentrated into 1000-fold through high speed concentration [70]. The final titer of the Lenti-TVA-mKate is 3×10^7^ infecting unit per milliliter.

The rabies virus (RV) and the cell lines for rabies propagation and tittering were kindly supplied by Callaway, E. M and prepared in our laboratory as previously described [40]. Briefly, RV-G pseudo typed SAD19-ΔG-mcherry was propagated in B7GG cells, and the supernatant was harvested with a titer of 105 infecting units/ml. To produce the EnvA-pseudotyped rabies, a Bhk-Enva cell was infected with filtered (0.45 μm, Millipore) RV-G-SAD19-ΔG-mcherry (EnvA-RV-mcherry). At six hours post infection, the Bhk-Enva cell were digested with 0.25% trypsin(Hyclone)to eliminate the contamination of RV-G pseudo typed rabies. During harvest of EnvA-RV-mcherry, the filtered supernatant was 2000–3000 fold concentrated through two cycles of high speed concentration as previous described [71]. The concentrated aliquots were tittered in 293 t-tva800 cell line. The final titer of EnvA-RV-mcherry was 2x108 infecting units per milliliter. All aliquots were stored at -80°C.

#### *Virus anterograde tracing*

Virus tracing works were performed in a BSL II animal facility. To label the ACC neurons, we first micro-injected 200 nl of the VSV-G pseudotyped Lenti-TVA-mKate into unilateral deep layers of the ACC as same as the Pha-L injection site. Four days post the infection of lentivirus, 400 nl of the EnvA-RV-mcherry was microinjected into the ACC. One week after rabies infection, mice were deeply anesthetized and transcardially perfused. Coronal brain slices containing the ACC and sagittal spinal slices containing cervical enlargement were cut with a thickness of 40 μm and collected serially. For immunohistochemistry of the spinal sections, the free-floating sections were washed in 0.1 M phosphate buffered saline (PBS) solution for 3 × 5 min, followed by an incubation with 10% normal goat serum in PBS solution for 1–1.5 hour. Sections were then incubated overnight in a rabbit polyclonal anti-RFP (Abcam, ab62341, 1:500) followed with an FITC conjugated anti-rabbit serum (1:200) for 4 hour at 4°C.

For fluorescent imaging of the brain sections labeled by rabies virus or immunostained spinal sections, the sections were washed with PBS, and wet mounted directly on Vecta-Shield mounting medium (brain sections were counterstained with DAPI), sealed with nail polish, imaged with an upright fluorescence confocal microscopy (Leica TCS SP8).

### Immunohistochemistry for Fos and FG

Seven days after making the nerve injury model, mice were anaesthetized and perfused with 0.1 mol/L PBS (pH 7.2–7.4) via the ascending aorta followed by 4% paraformaldehyde in 0.1 M PB (pH 7.4). The spinal cord and brain were then removed, and cryoprotected in 0.1 M PB containing 30% sucrose overnight at 4°C. Transverse sections (30 μm thickness) of spinal cord and brain samples were cut on a freezing microtome and collected serially and seperated as three sets of sections. Sections containing cervical spinal cord and ventral striatum were collected for injection sites imaging.

One set of sections containing ACC was used for Fos and FG immunostaining according to our previous works [21,72]. In brief, sections were sequentially incubated with the following solutions: (1) PBS solution of 3% bovine serum, 0.3% Triton X-100 (PBS-TX) containing mouse antisera against Fos (1:500, ab11959, Abcam) and rabbit antisera against FG (1:500, AB153, Millipore) for 2 days at 4°C, (2) an Alexa-594 conjugated anti-mouse (1:200, Invitrogen) and Alexa-488 conjugated anti-rabbit (1:200, Invitrogen) antibody in PBS-TX for 24 hrs at 4°C. Sections were then rinsed in PBS, mounted onto glass slides, air dried, cover-slipped with a mixture of 50% (v/v) glycerin and 2.5% (w/v) triethylene diamine in 0.01 M PBS. The signals were visualized under confocal microscope (FV-1000; Olympus, Tokyo, Japan) under appropriate filter for Alexa-488 (excitation 495 nm; emission 519 nm) and Alexa -594 (excitation 590 nm; emission 617 nm). For obsevation of the FG/Fos neurons, a careful focusing through the thickness of all sections determined that the immunolabeling had penetrated the whole thickness of the sections and only the neuronal cell bodies with obvious light emission were counted. Since the light from some positive neurons might be too weak to detect, the numbers of Fos-ir neurons and/or FG labeled neurons in Tables 1 and 2 should be regarded as representing the minimum of the real positive neurons in the sections. In addition, to avoid possible double counting of positive neurons the sections were carefully moved across the stage and analyzed from left to right.

One set of sections containing ACC was used for FG immunostaining and Nissl counterstaining. Sections were sequentially induced with (1) rabbit antisera against FG (1:500) for 2 days at 4°C, (2) biotin conjugated goat anti-rabbit antibody (1:200, Millipore) for 24 hrs at 4°C, (3) ABC elite kit (1:100) for 2 hrs. Finally, the sections were reacted with 0.05 M Tris–HCl buffer (pH 7.6) containing 0.04% DAB (Dojin) and 0.003% H_2_O_2_ for visualizing FG-like immunoreactive neurons. Then the sections were mounted onto gelatin-coated glass slides and processed for standard Nissl staining.

Another set of sections were used for control staining. The primary antibodies were omitted or replaced with normal rabbit/mouse serum and the other procedures were the same as those for the first 2 sets of sections in all groups. No staining was observed on brain sections when the primary antibody was omitted or replaced from the protocol.

### Whole-cell patch-clamp recordings

Coronal brain slices (300 μm) at the level of the ACC were prepared using standard methods^1,9,11^. Slices were transferred to a submerged recovery chamber containing oxygenated (95% O_2_ and 5% CO_2_) ACSF (124 mM NaCl, 4.4 mM KCl, 2 mM CaCl_2_, 1 mM MgSO_4_, 25 mM NaHCO_3_, 1 mM NaH_2_PO_4_, and 10 mM glucose) at room temperature for at least 1 hr and then heated up to 32°C for recording. Evoked EPSCs were recorded from layer V neurons in randomly selected sides of the ACC, with an Axon 200B amplifier, and the stimulations were delivered by a bipolar tungsten stimulating electrode placed in layer II/III of the ACC. AMPA receptor-mediated EPSCs were induced by repetitive stimulations at 0.02 Hz, and neurons were voltage-clamped at -60 mV in the presence of AP5 (50 μM). The recording pipettes (3–5 MΩ) were filled with a solution containing (in mM) 112 Cs-Gluconate, 5 TEA-Cl, 3.7 NaCl, 0.2 EGTA, 10 HEPES, 2MgATP, 0.3 Na_3_GTP and 5 QX-314 (adjusted to PH 7.2 with CsOH, 290 mOsmol). 0.1 mM spermine was included into the solution when recording AMPA I-V curve. Picrotoxin (100 μM) and AP5 (50 μM) was always present to block γ-aminobutyric acid (A) (GABA_A_) and NMDA receptor mediated synaptic currents in all experiments. To test the miniature EPSC, tetrodotoxin (1 mM) was added into the ACSF. The initial access resistance was 15–30 MΩ, and it was monitored throughout the experiment. Data were discarded if the access resistance changed >15% during experiment. Data were filtered at 1 kHz, and digitized at 10 kHz.

For recording ACC-spinal cord or ACC-ventral striatum projecting neurons, the ACC sections were observed under FV-1000 confocal microscope under proper filters for DiI (excitation 549 nm; emission 565 nm) or Alexa-488 Dextran Amine. In some cases, Alexa-488 or Alexa-594 was introduced into the recording solution for dual-labeling of the DiI or Dextran retrograde labeled neurons, respectively.

### Statistical analyses

All experiments were carried out as blind to genotype and the conditions of the experiments. Data were collected and processed randomly, and no data points were excluded. No statistical methods were used to predetermine sample sizes, but our sample sizes were similar to those reported in previous publications. Statistical comparisons were made using the unpaired, paired *t*-test, or two-way ANOVA (Tukey test was used for *post hoc* comparison). The normal distribution and the variation within each group of data was verified by using Sigmaplot 11.0 software before applying statistical comparison. Analyzed numbers (n) for each set of experiments are indicated in the corresponding figure legends or main text sections. The examples shown in each figure are representative and were reproducible at least three times for each set of experiments. All data were presented as the Mean ± S.E.M. In all cases, *p* < 0.05 was considered statistically significant.

## Abbreviations

ACC: Anterior cingulate cortex; ACSF: Artificial cerebrospinal fluid; AMPA: α-amino-3-hydroxy-5-methyl-4-isoxazolepropionic acid; APs: Action potentials; CPN: Common peroneal nerve; EPSCs: Excitatory postsynaptic currents; FG: Fluoro-Gold; GABA: Gamma-aminobutyric acid; I-O: Input-output; LTP: Long term potentiation; NMDA: N-Methyl-D-aspartate; PAG: Periaqueductal grey; PBS: Phosphate buffered saline; PKA: Protein kinase A; PKC: Protein kinase C; RV: Abies virus; RVM: Rostral medical medulla; SC: Spinal cord; TBS: Theta-burst stimulation; Thal: Thalamus; VS: Ventral striatum.

## Competing interests

The authors declare that they have no competing interests.

## Authors’ contributions

TC, YQL, and MZ designed the experiments. TC and KK performed the *in vitro* electrophysiological experiments. TC, SQ, JW, FW and LSZ carried out the immunostaining experiments. ZJZ, XBH, XQ, FQX and JH did the virus anterograde tracing experiments. RH provided the GluA1 phosphorylation site mutant mice and revised the manuscript. TC and MZ wrote the manuscript. All authors discussed the manuscript. All authors read and approved the final manuscript.
